# Contemporary Perspective on Addictive Behaviors: Underpinning Mechanisms, Assessment, and Treatment

**DOI:** 10.1155/2018/2069321

**Published:** 2018-06-05

**Authors:** Silvia Cimino, Carlos A. Almenara, Luca Cerniglia, Avinash Desousa, Angelo G. I. Maremmani

**Affiliations:** ^1^Department of Dynamic and Clinical Psychology, Faculty of Medicine and Psychology, Sapienza University of Rome, Rome, Italy; ^2^Universidad Peruana de Ciencias Aplicadas (UPC), Lima, Peru; ^3^Masaryk University, Czech Republic; ^4^Uninettuno Telematic International University, Rome, Italy; ^5^Lokmanya Tilak Municipal General Hospital and Lokmanya Tilak Municipal Medical College, Mumbai, India; ^6^Department of Psychiatry, North-Western Tuscany Region Local Health Unit, Versilia Zone, Viareggio, Italy

This special issue gathered contributions from authors in the scientific community working on addictive behaviors. In particular, authors were solicited to relate about underpinning mechanisms, assessment protocols, and intervention programs that are currently proposed for substance abuse, Internet addiction, and other forms of problematic conducts in pediatric populations, adolescence, and adulthood. Most of the papers used a biopsychosocial model for the onset and maintaining of addictive behaviors and their comorbidities with other psychopathologies. Although the intent was accept contributions focused on all forms of addictive behaviors, this special issue is composed of four papers concerning problematic use of the web and two articles focusing on substance use. Of note, all papers addressed the developmental phases of childhood and adolescence.

In the paper by M. Fuchs et al. entitled “Pathological Internet Use—An Important Comorbidity in Child and Adolescent Psychiatry: Prevalence and Correlation Patterns in a Naturalistic Sample of Adolescent Inpatients” the authors assessed the prevalence of comorbid Pathological Internet Use in a sample of adolescent with psychiatric disorders with the aim of unveiling correlations between PIU and psychiatric comorbidities. They found a very high prevalence of PIU among psychiatric patients and, in particular, addictive Internet use and problematic Internet use were, respectively, 8 times and 3 times higher among adolescents with psychiatric disorders, compared to a matched control sample.

In the paper by D. Öztaş et al. entitled “Evaluation of Risk Factors Affecting Substance Use among Tenth-Grade Students” the authors aimed to assess the prevalence of substance use among a large population of students, evaluating their thoughts, attitudes, behaviors, and tendencies towards substance use. They found that being male, being over the age of 15, living in in low income family with separated mother and father, and not being content with life were among the most significant risk factors for substance use.

In the paper by G. Ballarotto et al. entitled “Adolescent Internet Abuse: A Study on the Role of Attachment to Parents and Peers in a Large Community Sample” the authors evaluated the Internet use and abuse, the adolescents' attachment to parents and peers, and their psychological profiles in a large community sample of adolescents. They found that adolescents' attachment to parents had a significant effect on their use of the web. Moreover, adolescents' psychopathological risk had a moderating effect on the relationship between attachment to mothers and Internet use.

In the paper by S. Cimino and L. Cerniglia entitled “A Longitudinal Study for the Empirical Validation of an Etiopathogenetic Model of Internet Addiction in Adolescence Based on Early Emotion Regulation” the authors aimed at verifying if early emotion regulation strategies were predictive of school-age children's internalizing and externalizing symptoms, which in turn fostered Internet addiction in adolescence. Their results confirmed these hypotheses and indicated a direct statistical link between the characteristics of emotion regulation strategies in infancy and IA in adolescence.

In the paper by A. Porreca et al. entitled “Emotional Availability, Neuropsychological Functioning, and Psychopathology: The Context of Parental Substance Use Disorder” the authors aimed at investigating how maternal neuropsychological functioning and psychopathology are associated with mother-child emotional availability (EA), in the context of parental substance use disorder. In the study sample, they found high rates of maternal neuropsychological impairments and psychopathology, and generally low levels of EA were revealed. Moreover, maternal neuropsychological functioning was significantly associated with mother-child EA.

In the paper by C. Trumello et al. entitled “Relationship with Parents, Emotion Regulation, and Callous-Unemotional Traits in Adolescents' Internet Addiction” the authors aimed to investigate the associations between relationship with parents, emotion regulation, and callous-unemotional traits with Internet addiction in a community sample of adolescents. They found that a low perceived maternal availability, high cognitive reappraisal, and high callousness appeared to be predictors of Internet addiction.

